# In Memory of Professor Fereydoun Malekzadeh

**Published:** 2012-12

**Authors:** S Malekzadeh, MR Soudi

**Figure:**
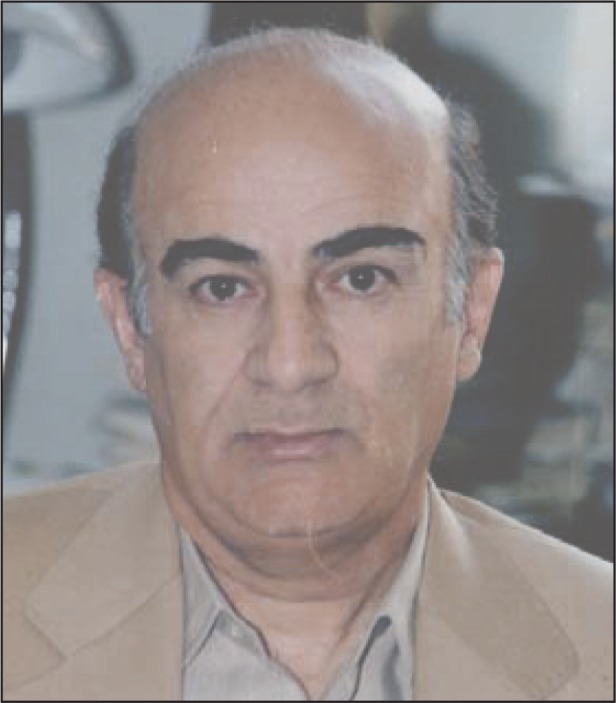
**Professor Fereydoun Malekzadeh**, one of the most accomplished microbiologists of Iran, transitioned on September 9, 2012, at the age of 79.

**Professor Fereydoun Malekzadeh** was born in 1933 (1312 AHS) in city of Tabriz in state of Azarbaijan in Iran. After graduating from high school, he migrated to Tehran to attend college. In 1956, he obtained his B.Sc. in *Biology* at Tehran University and was subsequently hired as an instructor in the faculty of Science at the same institution. In 1959 and after receiving his M.Sc. degree in *Mycology*, his high score in a nationwide test led to his nomination to receive a “Fulbright scholarship” to pursue his Ph.D. studies abroad. He chose to study *Bacteriology* at Louisiana State University (LSU). He completed his studies in less than 3 years and returned to his Alma mater to teach and conduct research as a young assistant professor. At 39 years of age, he became a full professor. Except for several Sabbaticals (University of Illinois, State University of Arizona, University of Göttingen, University of Alberta, Institute of Marine Biotechnology in University of Maryland) and until his semi-retirement in 2001, he taught continuously at Tehran University (and later, Azad University). Professor Feryedoun Malekzadeh was one of the main architects in establishing the Doctoral studies in the Microbiology program at Tehran University and Azad University. He played a great role in development of disciplines of *Microbiology* and related courses of academic studies in biology department of different Universities in Iran.

During his 46 years of active service as a faculty member in Faculty of Science at Tehran and Azad Universities, he authored and translated more than 36 books (a few are still used as reference text books) in Microbiology and Biological Sciences. *Introduction to Microbiology* is a familiar book among biology students, revised for the eight time and republished many times by different publishers. He authored numerous publications in peer-reviewed journals and lectured on various topics in microbiology and biotechnology. He received two “*Best Scientific Book of the Year*” award by Iranian presidents for his books (“*Botany*,” vol I & II; co-author: Dr. F. Moghadam, 1984) and “*Introduction to Microbiology*,” vol I&II, co-authour: Dr. M. Shahamat, 1990). In 1999, he was honored by receiving the “*UNESCO Science Prize*”, and “*International Kharazmi Award*” for discovering two new strains of bacteria. These two strains were then established in “Bergey's Manual of Systemic Bacteriology” as “*Cellulomonas persia*”, and “*Cellulomonas iranensis*”, and deposited in ATCC as reference strains. The most important part of his research focused on applied and environmental microbiology especially on biofilm and microbial bioremediation of pollutants. Even after his semi-retirement in 2001, he continued his contribution to the field of microbiology by writing and translating several scientific books. He was awarded the “Best Retired Researcher” at College of Sciences of Tehran University in 2004. He was one of the key founders of Iranian Society for Biology and a permanent member of numerous scientific bodies such as the “Academy of Persian Language and Literature” and the “American Society for Microbiology,” offering expertise and insight in optimal implementation of didactic and research venues.

An epitome of self-discipline, Dr. Malekzadeh's conduct demonstrated a high-caliber scientist with an analytical mind, an enthusiastic attitude in studying and pursuing new advances in microbiology and biotechnology and a tireless mentor in guiding and preparing the new generation of bright young scientists, thus, raising the bar in any institution of higher learning that employed his expertise.

He had a great love for Iran and the future of its youth. Despite ample employment opportunities outside Iran, he preferred to return to his Alma mater and share his enriched views after attending and presenting in many international symposiums. Diagnosed with a neurodegenerative disease in 2006, he stopped his academic activities and spent his last years of life with his family members and close friends.

He will be remembered by his immense scientific contributions to his homeland and the scientific community at large. His character, ethical standards, scientific contribution, teaching and training of thousands of young minds in the fields of medicine and science will help to continue the legacy of this exceptional scientist and honorable human being.

